# Decoding the Contribution of Shoulder and Elbow Mechanics to Barbell Kinematics and the Sticking Region in Bench and Overhead Press Exercises: A Link-Chain Model with Single- and Two-Joint Muscles

**DOI:** 10.3390/jfmk10030322

**Published:** 2025-08-20

**Authors:** Paolo Evangelista, Lorenzo Rum, Pietro Picerno, Andrea Biscarini

**Affiliations:** 1 Invictus Academy, 25126 Brescia, Italy; smartlifting@gmail.com; 2 Department of Engineering, University of Sassari, 07100 Sassari, Italy; lrum@uniss.it; 3 Department of Medicine and Surgery, University of Perugia, 06132 Perugia, Italy; andrea.biscarini@unipg.it

**Keywords:** biomechanics, bench press, sticking region, serial chain, modeling, muscle torque, joint load

## Abstract

**Objectives:** This study investigates the biomechanics of the bench press and overhead press exercises by modeling the trunk and upper limbs as a kinematic chain of rigid links connected by revolute joints and actuated by single- and two-joint muscles, with motion constrained by the barbell. The aims were to (i) assess the different contributions of shoulder and elbow torques during lifting, (ii) identify the parameters influencing joint loads, (iii) explain the origin of the sticking region, and (iv) validate the model against experimental barbell kinematics. **Methods:** Equations of motion and joint reaction forces were derived analytically in closed form. Dynamic simulations produced vertical barbell velocity profiles under various conditions. A waveform similarity analysis was used to compare simulated profiles with experimental data from maximal bench press trials. **Results:** The sticking region occurred when shoulder torque dropped below a critical threshold, resulting in a local velocity minimum. Adding elbow torque reduced this dip and shifted the velocity minimum from 38 cm to 23 cm above the chest, although it prolonged the time needed to overcome it. Static analysis revealed that grip width and barbell constraint had a greater effect on shaping the sticking region than muscle architecture parameters. Elbow extensors contributed minimally during early lift phases but became dominant near full extension. Model predictions showed high similarity to experimental data in the pre-sticking (SI = 0.962, *p* = 0.028) and sticking (SI = 0.949, *p* = 0.014) phases, with reduced, non-significant similarity post-sticking (SI = 0.881, *p* > 0.05) due to the assumption of constant torques. **Conclusions:** The model offers biomechanical insight into how joint torques and barbell constraints shape movement. The findings support training strategies that target shoulder strength early in the lift and elbow strength near lockout to minimize sticking and improve performance.

## 1. Introduction

Upper-limb strength exercises are fundamental in athletic preparation across a wide range of sports and are integral components of general fitness training routines [1]. Among these, multi-joint push exercises—such as the bench press and overhead press—are among the most frequently performed [2,3,4]. When executed with a free-weight barbell on a stable surface, these exercises allow for maximal force expression and are particularly popular among athletes and fitness enthusiasts. In contrast, performing them on an unstable base increases the activation of stabilizing muscles but reduces the involvement of primary movers [5,6]. Compared to dumbbells or resistance bands, barbells promote symmetrical movement by keeping the hands equidistant and leveraging their high moment of inertia around perpendicular axes, which helps counteract unwanted rotations. This allows lifters to focus more effectively on force production [7]. Unlike strength machines, a barbell enables the precise quantification of the lifted load, facilitating the objective monitoring of progress and comparisons against normative or peer data [8]. The mechanics of pressing machines—due to the inclusion of levers and cams—alter the force transmitted to the hand relative to the selected weight stack or plate load. These variations depend on machine design and require complex mechanical analyses to quantify accurately [9], precluding cross-machine comparisons or the establishment of universal normative values.

For these reasons, barbell-based multi-joint pressing exercises—especially the bench press—are widely regarded as the gold standard for evaluating upper-body strength [10] and have therefore been extensively studied. Research has focused on electromyographic patterns [11,12,13], barbell kinematics [14,15,16], and technical variations aimed at optimizing performance [17,18,19,20]. More recently, advanced biomechanical models of the bench press have been developed using OpenSim software (version 4.5) [21], incorporating all major contributing muscles and joint complexes, including the scapulothoracic joint. However, such models face limitations due to muscle redundancy, which requires optimization procedures that are often arbitrary and can reduce the reliability of the results [22]. Additionally, the use of generalized joint models with simplifying assumptions may neglect inter-subject variability, further affecting accuracy [23]. Due to their high complexity and extensive data requirements, these models have limited practical applicability and are less suited for addressing domain-specific questions, such as identifying the origin of the sticking region during barbell lifts [11,19,24,25,26,27,28,29,30]. The sticking region, defined as the portion of the lift in which barbell velocity decreases and reaches a local minimum [25,26,27], is a critical phase that can influence overall lift performance and provide insights into underlying neuromuscular or biomechanical limitations.

An alternative approach involves the use of analytical models based on simplified representations of the human body [31]. In this framework, the body is modeled as a kinematic chain of rigid links connected by actuated revolute joints. While simplistic, such models provide valuable insights by allowing for the investigation of cause–effect relationships between biomechanical parameters without the need for complex numerical simulations [31,32]. Furthermore, a single model can be adapted to multiple exercises. This modeling approach, common in robotics [33,34], has also been applied to the bench press to investigate specific aspects of performance [35,36,37]. However, these previous studies did not simulate barbell kinematics, nor did they explore the mechanisms underlying the sticking region, its morphology (i.e., pre-sticking, sticking, and post-sticking phases), or the time required to transition through them. Additionally, prior models often assumed that shoulder and elbow joints are driven by ideal torque generators, which produce joint torques without inducing reaction forces. Physiologically, however, these joints are actuated by muscles that span either a single joint (e.g., pectoralis major, anterior deltoid) or both joints (e.g., long head of triceps brachii, biceps brachii), producing not only torque but also tensile, compressive, and shear joint forces [38]. To accurately characterize the mechanical loading on joint structures, it is essential to adopt advanced models that simulate both single- and two-joint muscle actuators. Determining the individual contributions of muscle forces, external loads, and inertial effects to joint reaction forces remains one of the most complex and technically challenging tasks in musculoskeletal biomechanics [39]. To address these limitations, this present study analyzes multi-joint pushing exercises using a simplified model of the trunk and upper limbs, represented as a kinematic chain of rigid links connected by revolute joints and actuated by single- and two-joint muscles, with the barbell modeled as a mechanical constraint. We hypothesize that this model is capable of capturing the essential biomechanical characteristics of these exercises.

Specifically, we aim to:

• Explain the conditions that lead to the emergence of a sticking region during a lift.
• Highlight the respective contributions of shoulder and elbow torques to barbell kinematics across different phases of the movement.
• Identify the kinematic (joint angles, angular velocities, and accelerations) and dynamic (muscle forces, moments, external load, and barbell reaction forces) parameters that influence joint loads at the shoulder and elbow.
• Describe how the mechanical constraint imposed by the barbell affects all the above parameters.
• Reproduce the experimental barbell velocity patterns reported in the literature.

These insights, especially when validated against the experimental data, are intended to provide a rigorous and analytically grounded understanding of the biomechanical principles underlying multi-joint barbell pressing exercises. Such understanding offers practical guidance for researchers, coaches, rehabilitation professionals, and athletes seeking to optimize techniques and personalize training protocols for enhanced performance.

## 2. Materials and Methods

### 2.1. The Biomechanical Model

**Figure 1:**
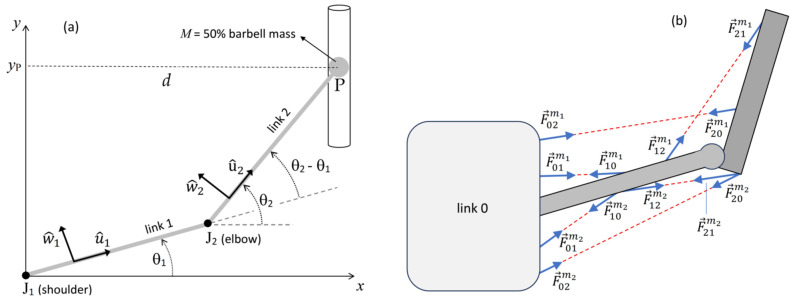
(**a**) Mechanical diagram of the two-link chain modeling a multi-joint barbell press exercise. The joints J_1_ and J_2_ represent the shoulder and elbow, respectively. To simulate the mechanical constraint imposed by the barbell, the chain endpoint is constrained to move along a straight vertical line, sliding along a frictionless vertical track. The proximal link (link 1) corresponds to the upper-arm segment, while the distal link (link 2) represents the combined forearm–arm segment and a concentrated mass located at the distal extremity of the link, equivalent to half the mass of the weighted barbell. The diagram includes the relevant angular quantities and unit vectors associated with each of the two links. (**b**) Schematic examples of single-joint muscle forces acting on link 0 ( ${\vec{F}}_{01}^{m_{1}}$, ${\vec{F}}_{01}^{m_{2}}$), link 1 ( ${\vec{F}}_{10}^{m_{1}}$, ${\vec{F}}_{10}^{m_{2}}$, ${\vec{F}}_{12}^{m_{1}}$, ${\vec{F}}_{21}^{m_{2}}$), and link 2 ( ${\vec{F}}_{21}^{m_{1}}$, ${\vec{F}}_{21}^{m_{2}}$), and two-joint muscle forces acting on link 0 ( ${\vec{F}}_{02}^{m_{1}}$, ${\vec{F}}_{20}^{m_{2}}$) and link 2 ( ${\vec{F}}_{20}^{m_{1}}$, ${\vec{F}}_{20}^{m_{2}}$). In the notation ${\vec{F}}_{ij}^{m}$, the subscript *i* denotes the link on which the force is exerted, *j* indicates the origin link of the muscle, and *m*_1_ and *m*_2_ identify different muscles.

We modeled the upper limbs and the barbell as two planar musculoskeletal chains, each consisting of two slender rigid links connected by ideal revolute joints, with a concentrated mass equal to half the barbell’s total mass incorporated at the distal extremity (endpoint P) of each chain (Figure 1a). The distal link of the chain (link 2) represents the combined forearm–hand segment and the concentrated mass, while the proximal link (link 1) corresponds to the upper-arm segment. Link 1 is articulated with link 2 at joint J_2_ and the chest—modeled as a fixed base (link 0)—at joint J_1_, which is also idealized as a revolute joint. To simulate the mechanical constraint imposed by the barbell on the chain, the endpoint of each chain has been constrained to move along a vertical straight line, sliding along a vertical frictionless track. Single-joint muscles (connecting link 1 to link 2, or link 1 to the base) and two-joint muscles (connecting link 2 to the base) are modeled as linear force actuators (Figure 1b). In line with a previous study [39], each of the two links (*i* = 1, 2) is characterized by the following parameters: mass $m_{i}$, length $l_{i}$, distance $l_{C_{i}}=\left|J_{i}C_{i}\right|$ from joint $J_{i}$ to the center of mass C*_i_* of the link, unit vector ${\hat{u}}_{i}$ pointing along the link from $J_{i}$, angle $\theta_{i}$ between ${\hat{u}}_{i}$ and the horizontal *x*-axis (with counterclockwise rotation considered positive), moment of inertia $I_{i}$ around $J_{i}$, angular velocity ${\vec{\omega }}_{i}={\dot{\theta }}_{i}\hat{k}$ and acceleration ${\dot{\vec{\omega }}}_{i}={\ddot{\theta }}_{i}\hat{k}$ ( $\hat{k}$ is the unit vector normal to the plane of the chain), and the unit vector ${\hat{w}}_{i}=\hat{k}\times {\hat{u}}_{i}$.

The external forces acting on link 1 include the link’s weight, $m_{1}\vec{g}$, applied at C_1_; the muscle forces $\sum_{m}{\vec{F}}_{12}^{\left(m\right)}$ and $\sum_{m}{\vec{F}}_{10}^{\left(m\right)}$ exerted on link 1 by the muscles connecting link 1 to link 2 and to link 0, respectively; and the joint reaction forces ${\vec{\phi }}_{10}$ and ${\vec{\phi }}_{12}$ exerted on link 1 by the base (link 0) and by link 2, respectively. The external forces acting on link 2 include the link’s weight, $m_{2}\vec{g}$, applied at C_2_; the muscle forces $\sum_{m}{\vec{F}}_{21}^{\left(m\right)}$ and $\sum_{m}{\vec{F}}_{20}^{\left(m\right)}$ exerted on link 2 by the muscles connecting link 2 to link 1 and to the base, respectively; the joint reaction force ${\vec{\phi }}_{21}$ exerted on link 2 by link 1; and the horizontal reaction force $R_{x}\hat{i}$ exerted by the vertical track on the endpoint ($R_{x}$ denotes the force component relative to the *x*-axis, and $\hat{i}$ is the x-axis unit vector). The adopted $J_{1}$*xy* global reference system is displayed in Figure 1a. The summations $\sum_{m}{\vec{F}}_{ij}^{\left(m\right)}$ extend over all muscles joining link *i* to link *j*, and $P_{ij}^{\left(m\right)}$ denotes the point of application of the muscle force ${\vec{F}}_{ij}^{\left(m\right)}$. The following relations hold: ${\vec{\phi }}_{ij}=-{\vec{\phi }}_{ji}$ and ${\vec{F}}_{ij}^{\left(m\right)}=-{\vec{F}}_{ji}^{\left(m\right)}$.

### 2.2. The Barbell Constraint

The constraint on the movement of the concentrated overload mass is analytically defined by the following equation:

(1) $$l_{1}\cos\theta_{1}\;+\;l_{2}\cos\theta_{2}=d$$

where *d* is the horizontal distance between J_1_ and the vertical line of endpoint movement (Figure 1a). Appendix A provides the relationships expressing the trigonometric function of $\theta_{2}$ in terms of $\theta_{1}$ (Equation (A1)). Differentiating Equation (1) with respect to $\theta_{1}$ yields:

(2) $$\frac{d\theta_{2}}{d\theta_{1}}=-\frac{l_{1}}{l_{2}}\;\frac{\sin\theta_{1}}{\sin\theta_{2}}$$

This equation, combined with Equation (A1), yields the following relationship:

(3) $${\dot{\theta }}_{2}\equiv \frac{d\theta_{2}}{dt}=\frac{d\theta_{2}}{d\theta_{1}}\frac{d\theta_{1}}{dt}=-\;\frac{l_{1}\sin\theta_{1}}{l_{2}\sin\theta_{2}}{\dot{\theta }}_{1}$$

which expresses ${\dot{\theta }}_{2}$ in terms of $\theta_{1}$ and ${\dot{\theta }}_{1}$. Similarly, differentiating this equation with respect to time expresses ${\ddot{\theta }}_{2}$ in terms of $\theta_{1}$ and its first- and second-time derivatives:

(4) $${\ddot{\theta }}_{2}=-\;\frac{l_{1}\sin\theta_{1}}{l_{2}\sin\theta_{2}}{\ddot{\theta }}_{1}-\;\frac{l_{1}\cos\theta_{1}}{l_{2}\sin\theta_{2}}{\dot{\theta }}_{1}^{2}-\;\frac{{\left(l_{1}\sin\theta_{1}\right)}^{2}l_{2}\cos\theta_{2}}{{\left(l_{2}\sin\theta_{2}\right)}^{3}}{\dot{\theta }}_{1}^{2}$$

### 2.3. Dynamic Equations of Link 2

The dynamic moment equation applied to link 2, which includes the overload mass, is given by:

(5) $$I_{2}{\ddot{\theta }}_{2}+J_{2}C_{2}\times m_{2}{\vec{a}}_{J_{2}}\cdot \hat{k}=\tau_{2}-m_{2}gl_{C_{2}}\cos\theta_{2}-R_{x}l_{2}\;\sin\theta_{2}$$

where $\tau_{2}$ is the axial torque around $J_{2}$ generated by forces exerted on link 2 by the single-joint muscles (originating from link 1) and two-joint muscles (originating from link 0) that insert at link 2. Alternatively, $\tau_{2}$ can be defined as the torque around $J_{2}\;$due to muscle forces acting on the links distal to joint $J_{2}\;$(i.e., only link 2 in a two-link chain), exerted by both the single-joint and two-joint muscles spanning joint $J_{2}$:

(6) $$\tau_{2}=\sum_{i}\left(J_{2}P_{21}^{\left(i\right)}\times {\vec{F}}_{21}^{\left(i\right)}\right)\cdot \hat{k}+\sum_{i}\left(J_{2}P_{20}^{\left(i\right)}\times {\vec{F}}_{20}^{\left(i\right)}\right)\cdot \hat{k}$$

Moments are considered positive when they generate angular acceleration in a counterclockwise direction. Consequently, positive values of $\tau_{2}$ indicate an elbow flexor torque, whereas negative values correspond to an elbow extensor torque. Considering the following relation (see Equation (A2) in Appendix A):

(7) $$J_{2}C_{2}\times m_{2}{\vec{a}}_{J_{2}}\cdot \hat{k}=l_{C_{2}}{\hat{u}}_{2}\times m_{2}\left({\ddot{\theta }}_{1}l_{1}{\hat{w}}_{1}-{\dot{\theta }}_{1}^{2}l_{1}{\hat{u}}_{1}\right)\cdot \hat{k}=m_{2}l_{C_{2}}l_{1}\left[{\ddot{\theta }}_{1}\cos\left(\theta_{2}-\theta_{1}\right)+{\dot{\theta }}_{1}^{2}\sin\left(\theta_{2}-\theta_{1}\right)\right]$$

Equation (5) can be rewritten as:

(8) $$\tau_{2}=m_{2}gl_{C_{2}}\cos\theta_{2}+R_{x}l_{2}\;\sin\theta_{2}+I_{2}{\ddot{\theta }}_{2}+m_{2}l_{C_{2}}l_{1}\left[{\ddot{\theta }}_{1}\cos\left(\theta_{2}-\theta_{1}\right)+{\dot{\theta }}_{1}^{2}\sin\left(\theta_{2}-\theta_{1}\right)\right]$$

which enables the determination of the horizontal reaction force $R_{x}$ that constrains the overload to vertical movement:

(9) $$R_{x}=\frac{1}{l_{2}\sin\theta_{2}}\left[\tau_{2}-{m_{2}gl}_{C_{2}}\cos\theta_{2}-I_{2}{\ddot{\theta }}_{2}-m_{2}l_{C_{2}}l_{1}\left[{\ddot{\theta }}_{1}\cos\left(\theta_{2}-\theta_{1}\right)+{\dot{\theta }}_{1}^{2}\sin\left(\theta_{2}-\theta_{1}\right)\right]\right]$$

The dynamic force equation of link 2, as seen here:

(10) $$m_{2}{\vec{a}}_{C_{2}}=m_{2}\vec{g}+\;R_{x}\hat{i}+\sum_{i}{\vec{F}}_{21}^{\left(i\right)}+\sum_{i}{\vec{F}}_{20}^{\left(i\right)}+{\vec{\phi }}_{21}$$

determines the joint reaction force ${\vec{\phi }}_{21}$ exerted by link 1 on link 2:

(11) $${\vec{\phi }}_{21}=m_{2}{\vec{a}}_{C_{2}}-m_{2}\vec{g}-\sum_{i}{\vec{F}}_{21}^{\left(i\right)}-\sum_{i}{\vec{F}}_{20}^{\left(i\right)}-\;\frac{1}{l_{2}\sin\theta_{2}}\left[\tau_{2}-{m_{2}gl}_{C_{2}}\cos\theta_{2}-I_{2}{\ddot{\theta }}_{2}-m_{2}l_{C_{2}}l_{1}\left[{\ddot{\theta }}_{1}\cos\left(\theta_{2}-\theta_{1}\right)+{\dot{\theta }}_{1}^{2}\sin\left(\theta_{2}-\theta_{1}\right)\right]\right]\hat{i}$$

where Equation (9) has been used.

### 2.4. Dynamic Equations of Link 1

The dynamic moment equation of link 1 is given by:

(12) $$I_{1}{\ddot{\theta }}_{1}=\sum_{i}\left(J_{1}P_{10}^{\left(i\right)}\times {\vec{F}}_{10}^{\left(i\right)}\right)\cdot \hat{k}+\sum_{i}\left(J_{1}P_{12}^{\left(i\right)}\times {\vec{F}}_{12}^{\left(i\right)}\right)\cdot \hat{k}-m_{1}gl_{C_{1}}\cos\theta_{1}+J_{1}J_{2}\times {\vec{\phi }}_{12}\cdot \hat{k}$$

Substituting the joint reaction force ${\vec{\phi }}_{12}=-{\vec{\phi }}_{21}$ from Equation (10) into Equation (12), we obtain:

(13) $$I_{1}{\ddot{\theta }}_{1}=\sum_{i}\left(J_{1}P_{10}^{\left(i\right)}\times {\vec{F}}_{10}^{\left(i\right)}\right)\cdot \hat{k}+\sum_{i}\left(J_{1}P_{12}^{\left(i\right)}\times {\vec{F}}_{12}^{\left(i\right)}\right)\cdot \hat{k}-m_{1}gl_{C_{1}}\cos\theta_{1}+l_{1}{\hat{u}}_{1}\times \left(-m_{2}{\vec{a}}_{C_{2}}+m_{2}\vec{g}+\;R_{x}\hat{i}\right)\cdot \hat{k}+\sum_{i}\left(J_{1}J_{2}\times {\vec{F}}_{21}^{\left(i\right)}\right)\cdot \hat{k}+\sum_{i}\left(J_{1}J_{2}\times {\vec{F}}_{20}^{\left(i\right)}\right)\cdot \hat{k}$$

Since $l_{1}{\hat{u}}_{1}\times m_{2}\vec{g}\cdot \hat{k}=-m_{2}gl_{1}\cos\theta_{1}$, $l_{1}{\hat{u}}_{1}\times R_{x}\hat{i}\cdot \hat{k}=-l_{1}R_{x}\sin\theta_{1}$, and we have the following (see Equation (A2) in Appendix A):

(14) $$J_{1}J_{2}\times m_{2}{\vec{a}}_{C_{2}}\cdot \hat{k}=l_{1}{\hat{u}}_{1}\times m_{2}\left({\ddot{\theta }}_{1}l_{1}{\hat{w}}_{1}-{\dot{\theta }}_{1}^{2}l_{1}{\hat{u}}_{1}+{\ddot{\theta }}_{2}l_{C_{2}}{\hat{w}}_{2}-{\dot{\theta }}_{2}^{2}l_{C_{2}}{\hat{u}}_{2}\right)\cdot \hat{k}=m_{2}l_{1}^{2}{\ddot{\theta }}_{1}+m_{2}{l_{1}l}_{C_{2}}\left[{\ddot{\theta }}_{2}\cos\left(\theta_{2}-\theta_{1}\right)-{\dot{\theta }}_{2}^{2}\sin\left(\theta_{2}-\theta_{1}\right)\right]$$

Equation (13) becomes:

(15) $$\begin{array}{r} I_{1}{\ddot{\theta }}_{1}=\sum_{i}\left(J_{1}P_{10}^{\left(i\right)}\times {\vec{F}}_{10}^{\left(i\right)}\right)\cdot \hat{k}+\sum_{i}\left(J_{1}P_{12}^{\left(i\right)}\times {\vec{F}}_{12}^{\left(i\right)}\right)\cdot \hat{k}-m_{1}gl_{C_{1}}\cos\theta_{1}-m_{2}gl_{1}\cos\theta_{1}-l_{1}R_{x}\sin\theta_{1}-m_{2}l_{1}^{2}{\ddot{\theta }}_{1}\\ -m_{2}{l_{1}l}_{C_{2}}\left[{\ddot{\theta }}_{2}\cos\left(\theta_{2}-\theta_{1}\right)-{\dot{\theta }}_{2}^{2}\sin\left(\theta_{2}-\theta_{1}\right)\right]+\sum_{i}\left(J_{1}J_{2}\times {\vec{F}}_{21}^{\left(i\right)}\right)\cdot \hat{k}+\sum_{i}\left(J_{1}J_{2}\times {\vec{F}}_{20}^{\left(i\right)}\right)\cdot \hat{k} \end{array}$$

The four muscle force moment summations can be rearranged as follows:

(16) ∑iJ1P10(i)×F→10(i)·k^+∑iJ1P12(i)×F→12(i)·k^+∑iJ1J2×F→21(i)·k^+∑iJ1J2×F→20(i)·k^      =∑iJ1P10(i)×F→10(i)·k^−∑iJ1P21i×F→21i·k^      +∑iJ1P21i×F→21i·k^−∑iJ2P21i×F→21i·k^      +∑iJ1P20i×F→20i·k^−∑iJ2P20i×F→20i·k^      =∑iJ1P10i×F→10i·k^+∑iJ1P20i×F→20i·k^      −∑iJ2P21i×F→21i·k^+∑iJ2P20i×F→20i·k^=τ1−τ2

where, according to Equation (6), the terms inside the second pair of brackets equal $\tau_{2}$, and $\tau_{1}$ is the “torque about $J_{1}$ due to muscle forces acting on the links distal to joint $J_{1}$ (i.e., on link 1 and link 2), exerted by both the single-joint and two-joint muscles spanning joint $J_{1}$”

(17) $$\tau_{1}=\sum_{i}\left(J_{1}P_{10}^{\left(i\right)}\times {\vec{F}}_{10}^{\left(i\right)}\right)\cdot \hat{k}+\sum_{i}\left({J_{1}P_{20}^{\left(i\right)}\times \vec{F}}_{20}^{\left(i\right)}\right)\cdot \hat{k}$$

Positive values of $\tau_{1}$ reflect a shoulder transverse flexor or abductor torque. Considering Equation (9) and Equation (16), Equation (15) takes its final form:

(18) $$\begin{array}{c} \tau_{1}=\tau_{2}\left(1+\frac{l_{1}\sin\theta_{1}}{l_{2}\sin\theta_{2}}\right)+m_{1}gl_{C_{1}}\cos\theta_{1}+m_{2}gl_{1}\cos\theta_{1}+\left(I_{1}+m_{2}l_{1}^{2}\right){\ddot{\theta }}_{1}+m_{2}{l_{1}l}_{C_{2}}\left[{\ddot{\theta }}_{2}\cos\left(\theta_{2}-\theta_{1}\right)-{\dot{\theta }}_{2}^{2}\sin\left(\theta_{2}-\theta_{1}\right)\right]\\ \;-\frac{l_{1}\sin\theta_{1}}{l_{2}\sin\theta_{2}}\left[{m_{2}gl}_{C_{2}}\cos\theta_{2}+I_{2}{\ddot{\theta }}_{2}+m_{2}l_{C_{2}}l_{1}\left[{\ddot{\theta }}_{1}\cos\left(\theta_{2}-\theta_{1}\right)+{\dot{\theta }}_{1}^{2}\sin\left(\theta_{2}-\theta_{1}\right)\right]\right] \end{array}$$

In the static condition, this equation simplifies to:

(19) $$\tau_{1}=\tau_{2}\left(1+\frac{l_{1}\sin\theta_{1}}{l_{2}\sin\theta_{2}}\right)+m_{1}gl_{C_{1}}\cos\theta_{1}+m_{2}gl_{1}\cos\theta_{1}-m_{2}gl_{C_{2}}\frac{l_{1}\sin\theta_{1}}{l_{2}\sin\theta_{2}}\cos\theta_{2}$$

Ideally, with no contribution of $\tau_{2}$, the shoulder transverse flexor (or abductor) torque $\tau_{1,eq}$ required to maintain the system in static equilibrium is:

(20) $$\tau_{1,eq}=m_{1}gl_{C_{1}}\cos\theta_{1}+m_{2}gl_{1}\cos\theta_{1}-m_{2}gl_{C_{2}}\frac{l_{1}\sin\theta_{1}}{l_{2}\sin\theta_{2}}\cos\theta_{2}$$

Conversely, with no contribution of $\tau_{1}$, the equilibrium elbow extensor torque $-\tau_{2,eq}$ is:

(21) $$-\tau_{2,eq}=\frac{\tau_{1,eq}}{1+\frac{l_{1}\sin\theta_{1}}{l_{2}\sin\theta_{2}}}=\frac{m_{1}gl_{C_{1}}\cos\theta_{1}+m_{2}gl_{1}\cos\theta_{1}-m_{2}gl_{C_{2}}\frac{l_{1}\sin\theta_{1}}{l_{2}\sin\theta_{2}}\cos\theta_{2}}{1+\frac{l_{1}\sin\theta_{1}}{l_{2}\sin\theta_{2}}}$$

The dynamic force equation of link 1, as seen here:

(22) $$m_{1}{\vec{a}}_{C_{1}}=m_{1}\vec{g}+\sum_{i}{\vec{F}}_{12}^{\left(i\right)}+\sum_{i}{\vec{F}}_{10}^{\left(i\right)}+{\vec{\phi }}_{10}+{\vec{\phi }}_{12}$$

determines the joint reaction force ${\vec{\phi }}_{10}$ acting on J_1_:

(23) $${\vec{\phi }}_{10}=m_{1}{\vec{a}}_{C_{1}}-m_{1}\vec{g}-\sum_{i}{\vec{F}}_{12}^{\left(i\right)}-\sum_{i}{\vec{F}}_{10}^{\left(i\right)}-{\vec{\phi }}_{12}$$

With the aid of Equation (11), this expression takes its final form:

(24) $${\vec{\phi }}_{10}=m_{1}{\vec{a}}_{C_{1}}+m_{2}{\vec{a}}_{C_{2}}-\left(m_{1}+m_{2}\right)\vec{g}-\sum_{i}{\vec{F}}_{10}^{\left(i\right)}-\sum_{i}{\vec{F}}_{20}^{\left(i\right)}-\frac{1}{l_{2}\sin\theta_{2}}\left[\tau_{2}-m_{2}gl_{C_{2}}\cos\theta_{2}-I_{2}{\ddot{\theta }}_{2}-m_{2}l_{C_{2}}l_{1}\left[{\ddot{\theta }}_{1}\cos\left(\theta_{2}-\theta_{1}\right)+{\dot{\theta }}_{1}^{2}\sin\left(\theta_{2}-\theta_{1}\right)\right]\right]\hat{i}$$

### 2.5. Numerical Simulation

In all the above equations, ${\ddot{\theta }}_{2}$, ${\dot{\theta }}_{2}^{2}$, $\sin\theta_{2}$, $\cos\theta_{2}$, $\sin\left(\theta_{2}-\theta_{1}\right)$, and $\cos\left(\theta_{2}-\theta_{1}\right)$ can be expressed as functions of $\theta_{1}$ and its first- and second-time derivatives using Equations (3), (4) and (A1). This is consistent with the fact that the system has one degree of freedom. Thus, the system configuration is conveniently referred to angle $\theta_{1}$ or the vertical position of the barbell—specifically, the vertical position $y_{P}$ of the chain endpoints, which can be expressed as a function of $\theta_{1}$:

(25) $$y_{P}=l_{1}\sin\theta_{1}+l_{2}\;\sin\theta_{2}=l_{1}\sin\theta_{1}+l_{2}\sqrt{1-{\left(\frac{d}{l_{2}}-\frac{l_{1}}{l_{2}}\cos\theta_{1}\right)}^{2}}$$

The lift is completed when the elbow is fully extended $\left(\theta_{2}=\theta_{1}=\arccos\left[d/\left(l_{1}+l_{2}\right)\right]\right)$, and the barbell reaches its final position ( $y_{P,max}=\sqrt{{\left(l_{1}+l_{2}\right)}^{2}-d^{2}}$).

With the use of Equations (19)–(21), we determined the required combinations of the torque of $\tau_{1,eq}$ and $\tau_{2,eq}$, normalized to the weight *Mg* of mass *M* (the concentrated mass at the endpoint of each chain equal to half of the barbell mass), which is necessary to maintain system equilibrium. By employing Equation (18), we analyzed the influence of $\tau_{1}$ and $\tau_{2}$ (normalized to *Mg*) on the system kinematics for different values of *d* (which determines the grip width) and the initial system configuration, determined by the initial value $\theta_{1}(0)$ of $\theta_{1}$. Specifically, two conditions were examined to simulate the barbell bench press (d=25 cm, $\theta_{1}\left(0\right)=-45{}^{\circ}$, M=50 kg) and the overhead press (d=10 cm, $\theta_{1}\left(0\right)=-60{}^{\circ}$, M=33.3 kg). These values of *M* were selected based on weightlifting strength standards for intermediate-level users [40]. However, the study results were largely independent of *M*, since $\tau_{1}$ and $\tau_{2}$ were normalized to *Mg*, and *M* is significantly greater than the masses of the forearm and upper-arm. The anthropometric and inertial parameters ($l_{1},\;l_{2},\;l_{C_{1}},l_{C_{2}},\;m_{1},\;m_{2},\;I_{1},I_{2}$) were obtained from the relevant scientific literature [32,41], taking into account the concentrated mass *M* in the determination of $l_{C_{2}}$ and $m_{2}$, in accordance with the model definition. Numerical simulations were carried out using the Maple software package (2024). Finally, the joint loads acting on the shoulder and elbow were qualitatively assessed using Equations (11) and (24).

### 2.6. Experimental Data

The validity of the model in predicting barbell velocity was assessed against the experimental data. The velocity–time curves used for validation were obtained from a previously published study [42], in which barbell velocity during a one-repetition maximum (1RM) bench press test performed by seven elite-level international Paralympic powerlifters was determined through photogrammetric analysis. Given that one of the goals of the model is to describe the mechanics of the sticking phase, we focused on waveform similarity. To achieve this, we applied Constrained Dynamic Time Warping (cDTW) [43] after normalizing both signals to 100% of their length. DTW was then applied separately to three predefined regions based on characteristic landmarks of the experimental time series [11,19,24,25,26,27,28,29,30]. These regions were segmented using three anchor points: the pre-sticking region (from the start to the first peak), the sticking region (from the first peak to the velocity minimum), and the post-sticking region (from the velocity minimum to the end of the motion). Local window constraints (*w*) were empirically determined to find a balance between trajectory shape preservation and overfitting avoidance and were set to *w* = 20 (i.e., 20% of the signal segment length) [44]. The cDTW similarity index (SI), ranging from 0 (no similarity) to 1 (full similarity), was computed for each segment as follows:

(26) $$SI=\frac{1-D}{D+L}$$

where *D* is the cDTW distance and *L* is the length of the longest time series [43]. To assess statistical significance, we performed a permutation test where the experimental and model time series were randomly shuffled 1000 times, and the cDTW similarity was recalculated for each permutation [45]. The *p*-value was defined as the proportion of permutations yielding a similarity index greater than or equal to the observed value. This test was performed both globally and for each predefined region, following the approach commonly used in time-series analysis [46].

## 3. Results

### 3.1. Kinematics

**Figure 2:**
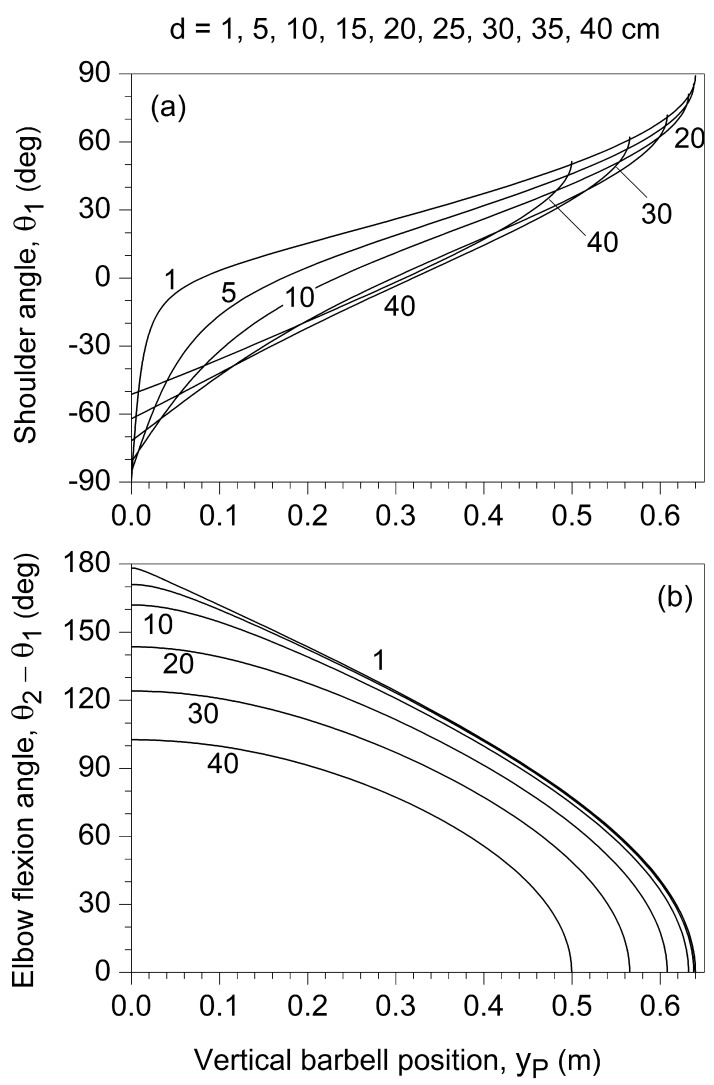
Dependence on the vertical barbell position ($y_{P}$) of the shoulder angle $\theta_{1}$ (**a**) and the elbow flexion angle $\theta_{2}-\theta_{1}$ (**b**) for different values of *d.* The parameter *d* represents the horizontal distance between the shoulder (J_1_) and the vertical line of movement of the endpoint (P).

The shoulder transverse-flexion/abduction angle, $\theta_{1}$ ( $\theta_{1}=0$ when link 1 is horizontal), increased nonlinearly with $y_{P}$ and was significantly influenced by *d* only at the lower values of *d* and $y_{P}$ (Figure 2a). Conversely, the elbow flexion angle, $\theta_{2}-\theta_{1}$, decreased nonlinearly with a progressively higher rate as either $y_{P}$ or *d* increased (Figure 2b).

### 3.2. Static Analysis

**Figure 3:**
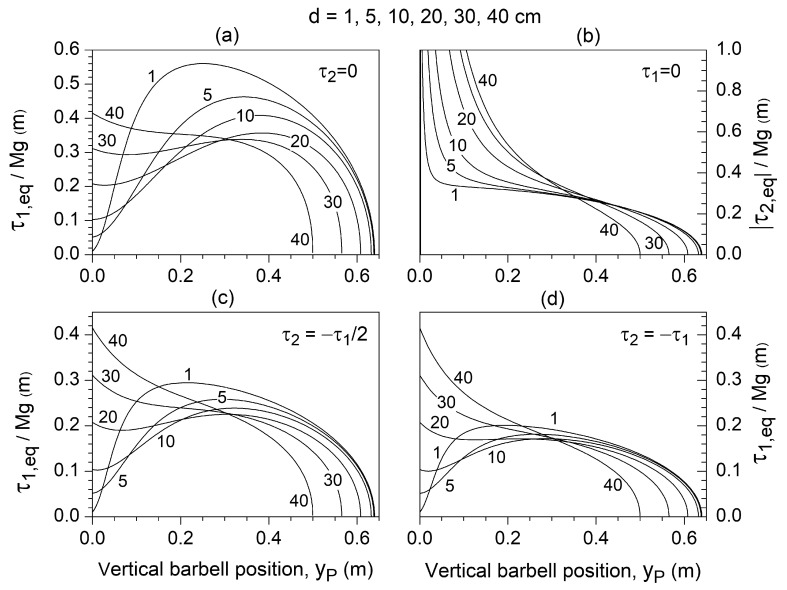
(**a**) Dependence of the shoulder transverse flexor torque needed to maintain the system in equilibrium ( $\tau_{1,eq}$) at the vertical barbell (i.e., endpoint) position $y_{P}$ for different values of *d*, assuming negligible elbow extensor torque $\left(\tau_{2}=0\right).$ (**b**) Dependence of the elbow extensor torque needed to maintain the system in equilibrium ($\tau_{2,eq}$) at the vertical barbell position $y_{P}$ for different values of *d*, assuming a negligible shoulder transverse flexor torque $\left(\tau_{1}=0\right).$ Dependence of the shoulder transverse flexor torque needed to maintain the system in equilibrium ($\tau_{2,eq}$) on the vertical barbell position $y_{P}$ for different values of *d*, assuming that the elbow extensor torque equals 50% (**c**) and 100% (**d**) of the transverse shoulder flexor torque ($\tau_{2}=-\tau_{1}/2$ and $\tau_{2}=-\tau_{1}$, respectively). Torque $\tau_{1,eq}$ and $\tau_{2,eq}$ are normalized to the weight *Mg* of the concentrated mass applied at the chain endpoint, which equals half of the weight of the loaded barbell.

In static conditions, if the torque $\tau_{2}$ were negligible, the muscle torque developed around joint J_1_ needed to maintain the system in equilibrium ( $\tau_{1,eq}$) would be strongly dependent on *d*, which represents the grip width (Figure 3a). The grip width can be expressed as approximately 2*d* + *d*_s_, where *d*_s_ is the biacromial distance. For *d* greater than d* = 37 cm, $\tau_{1,eq}$ is a non-linear descending function of $y_{P}$. As *d* is gradually reduced below this threshold, $\tau_{1,eq}$ reaches a relative maximum whose magnitude progressively increases, with its position initially shifting from $y_{P}=$ 17 cm to about $y_{P}=38$ cm before returning towards $y_{P}=25$ cm as *d* reaches 1 cm. Conversely, if $\tau_{1}$ were negligible, the equilibrating elbow extensor torque $\tau_{2,eq}$ would always behave as a descending function of $y_{P}$, with its behavior also depending nonlinearly on *d* (Figure 3b).

**Figure 4:**
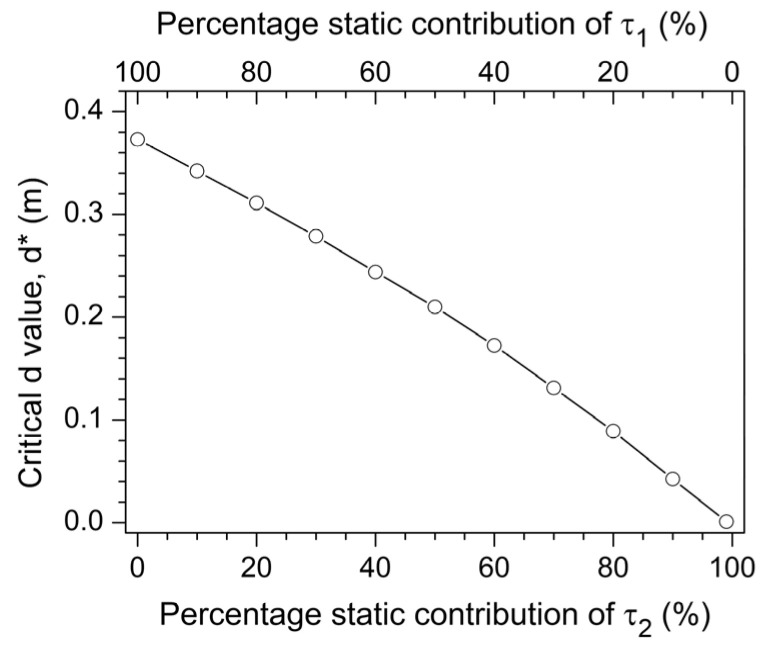
Dependence of the critical value d* on the percentage of static contribution from $\tau_{1}$ and $\tau_{2}$ (% $\tau_{1}$ and % $\tau_{2}$, with % $\tau_{1}+\%\tau_{2}=100$). The parameter d* represents the horizontal distance between the shoulder (J_1_) and the vertical line of movement of the endpoint (P), below which a local maximum occurs in the equilibrating torques, as reported in Figure 3.

In realistic scenarios, both $\tau_{1}$ and $\tau_{2}$ contribute to the system’s equilibrium. Given that the transverse shoulder flexor torque [47] is approximately twice the elbow extensor torque [48], we examined this specific combination ($\tau_{2}=-\tau_{1}/2$) to analyze the bench press exercise (Figure 3c). Compared to the curves observed for $\tau_{2}=0$ (Figure 3a), the main effect of $\tau_{2}$, set as equal to $-\tau_{1}/2$, was to reduce the threshold d* to 27 cm and the magnitude of the relative maxima by approximately 40% (Figure 3c). Values of $\left|\tau_{2}\right|$ progressively higher (or lower) than $\tau_{1}/2$ resulted in correspondingly greater (smaller) reductions in d* and the relative maxima. For example, as $\tau_{2}=-\tau_{1}$ (Figure 3d), d* reduces to 21 cm, and the magnitude of the relative maxima displays a limited variation of about 0.19 $\tau_{1}/Mg$ with a further decrease of *d*. As the shoulder abductor torque is about three to two times greater than the elbow extensor torque [48,49], the overhead press exercise roughly corresponds to the condition $\tau_{2}=-2\tau_{1}/3$, which is intermediate between those displayed in Figure 3c,d. The decreasing trend of d* with the progressive higher percentage contribution of $\tau_{2}$ is highlighted in Figure 4.

### 3.3. Dynamic Analysis

**Figure 5:**
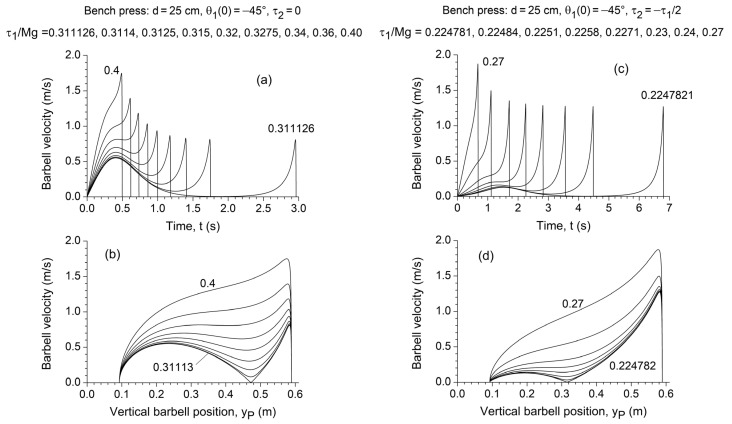
Dependence of the vertical barbell (i.e., endpoint) velocity ${\dot{y}}_{P}$ on time (**a**,**c**) and vertical barbell position $y_{P}$ (**b**,**d**) for d=25 cm, $\theta_{1}\left(0\right)=-45{}^{\circ}$, M=50 kg, and the different values of shoulder torque $\tau_{1}$, assuming a negligible elbow torque $\tau_{2}=0$ (**a**,**b**) and an elbow extensor torque $\tau_{2}=-\tau_{1}/2$ (**c**,**d**). $\tau_{1}$ is normalized to the weight *Mg* of the concentrated mass applied at the chain endpoint, which corresponds to half of the weight of the loaded barbell.

Under simulated bench press conditions (d=25 cm, $\theta_{1}\left(0\right)=-45{}^{\circ}$, $9\;cm\leq y_{P}\leq 59\;cm$), and in the absence of elbow torque contribution ($\tau_{2}=0$), the velocity ${\dot{y}}_{P}$ exhibits a monotonic increase with both $y_{P}$ and *t* when $\tau_{1}$ exceeds the critical threshold $\tau_{1}^{*}=0.34Mg$ (Figure 5a,b). Below this threshold, ${\dot{y}}_{P}$ follows a pattern characterized by a local maximum of 0.6 m/s at $y_{P}=$ 24 cm, followed by a local minimum around $y_{P}=$ 47 cm, with the velocity progressively approaching zero as $\tau_{1}$ is reduced to 0.3112·*Mg*. With the introduction of an elbow extensor torque $\tau_{2}=-\tau_{1}/2$, the transition occurs at $\tau_{1}=$0.2271·*Mg*, shifting the local maximum of ${\dot{y}}_{P}$ to $y_{P}=$ 19 cm with a reduced peak velocity of 0.15 m/s, while the local minimum moves to approximately $y_{P}=$ 32 cm (Figure 5c,d).

**Figure 6:**
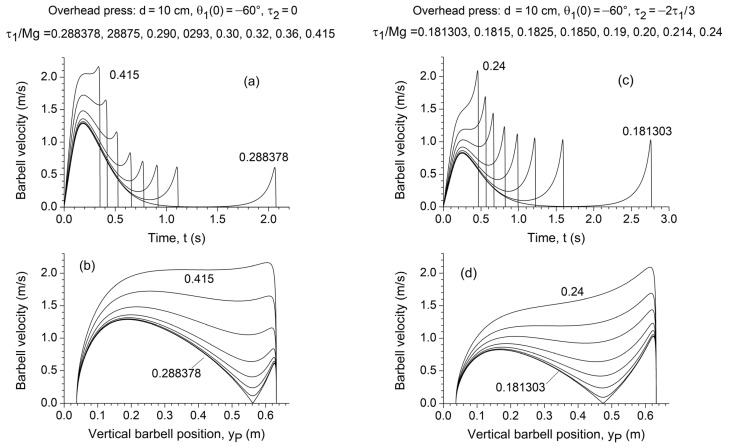
Dependence of the vertical barbell (i.e., endpoint) velocity ${\dot{y}}_{P}$ on time (**a**,**c**) and vertical barbell position $y_{P}$ (**b**,**d**) for d=10 cm, $\theta_{1}\left(0\right)=-60{}^{\circ}$, M=33.3 kg, and different values of shoulder torque $\tau_{1}$, assuming a negligible elbow torque $\tau_{2}=0$ (**a**,**b**) and an elbow extensor torque $\tau_{2}=-2\tau_{1}/3$ (**c**,**d**). $\tau_{1}$ is normalized to the weight *Mg* of the concentrated mass applied at the chain endpoint, which corresponds to half of the weight of the loaded barbell.

In the simulated overhead press conditions (d=10 cm, $\theta_{1}\left(0\right)=-60{}^{\circ}$, $4\;cm\leq y_{P}\leq 63\;cm$), similar trends are observed, albeit with notable differences. In the absence of elbow torque ($\tau_{2}=0$), local extrema emerge when $\tau_{1}<\tau_{1}^{*}=0.415$ *Mg* (Figure 6a,b). The local maximum, reaching approximately 1.3 m/s, appears at $y_{P}=$ 19 cm, while the local minimum approaches zero near full elbow extension ($y_{P}=$ 56 cm) as $\tau_{1}$ decreases towards 0.28838·*Mg*. When an elbow extensor torque $\tau_{2}=-2\tau_{1}/3$ is applied in addition to $\tau_{1}$, the critical threshold shifts to $\tau_{1}^{*}=$ 0.214·*Mg*, reducing the local maximum to 0.8 m/s and shifting the local minimum to $y_{P}=$ 47 cm (Figure 6c,d).

Notably, in both exercises, triceps involvement shifted the sticking region to an earlier phase of the lift and reduced its depth and extent (Figure 5d and Figure 6d). However, it also substantially increased the time required to overcome the sticking region, as well as the duration of the lift in both the pre- and post-sticking phases (Figure 5c and Figure 6c).

### 3.4. Comparison with Experimental Data

**Figure 7:**
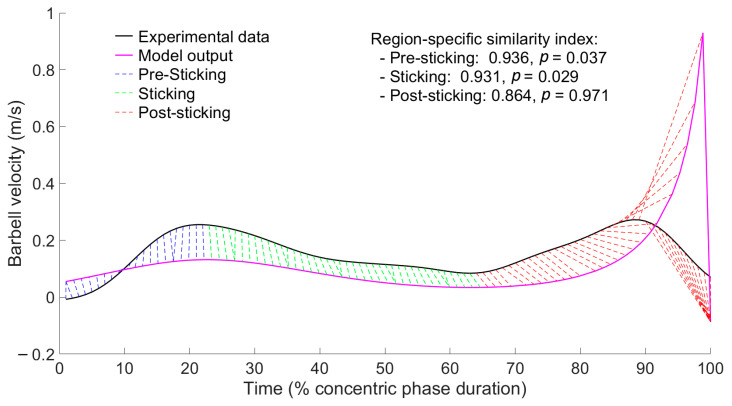
Waveform similarity analysis comparing barbell velocity patterns between experimental data from Rum et al. [42] for a 1 RM lift relative to a sample subject and the model outcome obtained for a simulated barbell bench press (d=25 cm, $\theta_{1}\left(0\right)=-45{}^{\circ}$, $\tau_{2}=-\tau_{1}/2$) with $\tau_{1}/Mg=0.22484$, a condition corresponding to a maximal lift.

The results relative to waveform similarity analysis (Figure 7) are presented as the median (IQR). A waveform similarity assessment with respect to experimental data from Rum et al. [42] for a 1 RM lift revealed a high and statistically significant similarity index for the pre-sticking (SI: 0.962 (0.019), p: 0.028 (0.033)) and sticking regions (SI: 0.949 (0.038), p: 0.014 (0.02)), while a lower and not statistically significant similarity was found for the post-sticking region (SI: 0.881 (0.012), p: 0.958 (0.205)). The comparison was performed between these experimental data and the model outcome obtained by simulating the barbell bench press (d=25 cm, $\theta_{1}\left(0\right)=-45{}^{\circ}$, $\tau_{2}=-\tau_{1}/2$) with $\tau_{1}/Mg=0.22484$, a condition corresponding to a maximal lift.

## 4. Discussion

This study analyzed the biomechanics of the barbell bench press and overhead press, modeling the trunk and upper limbs as a kinematic chain of rigid links connected by revolute joints, actuated by single- and two-joint muscles, and constrained by the barbell. The system equations of motion and the reaction forces acting on the shoulder and elbow were derived analytically in a closed form, quantifying the unique contribution of each relevant biomechanical parameter (Equations (11), (18) and (24)). Both static and direct dynamic analyses explained the condition for the appearance of a sticking region during the lift and clarified the differential contribution of shoulder and elbow torque across various lift phases.

The constraint imposed by the barbell plays a crucial role in shaping both the kinematics and dynamics of the system. This constraint induces a horizontal force $R_{x}\hat{i}$ (Equation (9)) at the distal end of the chain, generating an axial moment at these two joints. Notably, $R_{x}$ is not only influenced by $\tau_{2}$ and $m_{2}$ but also depends on the instantaneous values of the kinematic parameters of the two links.

The static analysis (Figure 3) highlighted how the distinct contributions of shoulder and elbow torque ($\tau_{1,eq}$ and $\tau_{2,eq}$) vary to maintain equilibrium throughout the different phases of the lift. These contributions are critically modulated by the key geometric parameter *d* (the horizontal distance between the shoulder joint J_1_ and the vertical path of the endpoint P), which ultimately determines grip width.

Assuming a negligible elbow torque ($\tau_{2}=0$), the equilibrating shoulder torque $\tau_{1,eq}$ exhibits a progressively increasing local maximum ${\left(\tau_{1,eq}\right)}_{max}$ when *d* is progressively reduced below a critical threshold (d* = 37 cm) (Figure 3a). In this condition, the application of a constant torque $\tau_{1}$ with a magnitude greater than the initial value of $\tau_{1,eq}$ but lower than ${\left(\tau_{1,eq}\right)}_{max}$ will lead to an initial acceleration phase ($\tau_{1}>\tau_{1,eq}$) followed by a deceleration phase ($\tau_{1}<\tau_{1,eq}$). If the barbell velocity drops to zero before surpassing the deceleration phase, the lift fails. Conversely, if the barbell reaches a height beyond the local maximum of $\tau_{1,eq}$, where the condition $\tau_{1}>\tau_{1,eq}$ is restored, the velocity continues increasing, allowing for the lift to be completed. The final outcome is a local minimum in the barbell velocity ${\dot{y}}_{P}$, corresponding to the sticking region. On the other hand, when the shoulder torque is assumed negligible ($\tau_{1}=0$), the equilibrating elbow extension torque $\tau_{2,eq}$ progressively decreases throughout the lift, regardless of *d* (Figure 3b). This indicates that the contribution of the elbow extensor muscles becomes increasingly effective in supporting the barbell, preventing any local minimum in ${\dot{y}}_{P}$ once a constant torque $\tau_{2}$ capable of initiating the movement is applied.

When both shoulder and elbow torques are applied simultaneously, intermediate conditions arise (Figure 3c,d). The elbow torque contributes minimally during the initial phase of the lift ($\tau_{1,eq}$ remains nearly independent of $\tau_{2,eq}$) but becomes increasingly significant in the final phase ($\tau_{1,eq}$ progressively decreases as $\tau_{2,eq}$ increases). Notably, the critical value d* associated with the appearance of a sticking region decreases progressively as the contribution of the elbow’s extensor torque compensates more for shoulder torque decrement (Figure 4). If $\tau_{1}$ and $\tau_{2}$ vary during the lift, the ranges at which acceleration and deceleration occur depend on their comparison with the corresponding equilibrium values $\tau_{1,eq}$ and $\tau_{2,eq}$. However, the relative changes in the $\tau_{1,eq}$ and $\tau_{2,eq}$ curves throughout the lift (Figure 3) largely exceed the variation in the maximum torque-generating capacity of the elbow and shoulder muscles within the same joint range of motion [50]. Consequently, the influence of variations in such maximum torque-generating capacities is expected to shift the transition points between acceleration and deceleration phases only marginally. This supports the study’s conclusion that the occurrence of a relative minimum in barbell velocity, namely the sticking region, is primarily determined by the system’s geometry and constraints, which determine the shape of the equilibrating torque curves $\tau_{1,eq}/Mg$ and $\tau_{2,eq}/Mg$ (Figure 3). These factors appear to be significantly more influential than the muscle architecture parameters that govern variations in the maximum torque-generating capacity of the elbow and shoulder muscles.

The results of the direct dynamic simulation further confirmed the predictions of the static analysis. For the bench press (d=25 cm, $\theta_{1}\left(0\right)=-45{}^{\circ}$, $9\;cm\leq y_{P}\leq 59\;cm$; Figure 5) and the overhead press (d=10 cm, $\theta_{1}\left(0\right)=-60{}^{\circ}$, $4\;cm\leq y_{P}\leq 63\;cm$; Figure 6), in the absence of the elbow torque contribution, a progressively wider and deeper local minimum appeared in the ${\dot{y}}_{P}(t)$ and ${\dot{y}}_{P}(y_{P})$ curves as the input shoulder torque $\tau_{1}$ was decreased below a critical value (Figure 5a,b and Figure 6a,b). The concurrent application of an input elbow extensor torque (set at $\tau_{2}=-\tau_{1}/2\;$for the bench press and $\tau_{2}=-2\tau_{1}/3$ for the overhead press) significantly reduced both the critical values of $\tau_{1}$, below which the local minimum occurred or the lift failed (Figure 5c,d and Figure 6c,d). Additionally, it shifted the local minimum of ${\dot{y}}_{P}$ to an earlier stage of the lift (lower value of $y_{P}$) while reducing the peak barbell velocity before the sticking region, ultimately shifting the sticking region earlier and reducing its depth and extent in the ${\dot{y}}_{P}(y_{P})$ plot. Nevertheless, it also substantially increased the time needed to overcome the sticking region, increasing its extension in the ${\dot{y}}_{P}(t)$ plot.

### 4.1. Practical Applications

The analytical framework developed in this study provides actionable insights for strength and conditioning professionals aiming to optimize performance and reduce injury risk in pressing barbell-constrained exercises. Specifically, the model indicates that the parameter *d*, which determines grip width (approximately 2*d* plus the inter-acromial distance), critically affects the torque balance between the shoulder and elbow and can determine the occurrence and location of the sticking region. Reducing *d* below the critical value d* increases shoulder torque demands and may shift the point of the local minimum of barbell velocity later in the lift, whereas increasing d or enhancing the elbow contribution can shift it earlier and reduce its depth. Coaches can exploit this by modulating parameter d according to the athlete’s sticking region profile—for example, slightly narrower grips to emphasize triceps contribution for late sticking regions or wider grips to enhance pectoral involvement for early sticking regions [26,29]. The simulations also suggest that modifying a joint’s starting angles can influence torque availability in the critical phase. Athletes with early sticking regions may benefit from a slightly more extended elbow at lift-off, while those with later sticking regions may benefit from maximizing scapular retraction to support shoulder torque. Complementary strategies consistent with the torque patterns predicted by the model include pressing from pins or boards at the height of the local minimum of barbell velocity and using variable resistance to better match torque demands across the range of motion [26,29].

### 4.2. Comparison with Experimental Data

These above practical considerations are further supported by the strong qualitative similarity between the model’s predictions and the velocity patterns recorded during flat bench presses performed by powerlifters with increasing loads [26,27,29,30,42,51,52,53,54], with the exception of the final phase of the lift, where the lower similarity was expected due to the model’s assumption of constant shoulder and elbow muscle torque throughout the entire movement. Notably, the bench press model with $\tau_{2}=-\tau_{1}/2\;$predicts that during a lift with a theoretical maximum load, the velocity reaches a local maximum of about 0.2 m/s before the sticking region, and the local minimum in barbell velocity occurs slightly before the midpoint of the lift (23 cm above the chest). These values closely match those experimentally measured in previous studies with maximal loads [30,51,52]. In the theoretical model, unlike the experimental case, the velocity at the local minimum is infinitesimally greater than zero, as would be expected in theory for a maximal load. The quantitative comparison between the predicted barbell velocity patterns (Figure 5c) and the experimental data from Rum et al. [42] fully supports these qualitative assessments. Waveform similarity analysis confirmed that the model accurately predicts both the pre-sticking and sticking regions, which aligns with its intended purpose (Figure 7), while showing lower and statistically non-significant similarity in the post-sticking region. This discrepancy in the post-sticking region was expected, as the model assumes a constant muscle torque at the shoulder and elbow throughout the lift, up to full elbow extension. Consequently, once the local minimum is surpassed, the barbell velocity continues to increase until full elbow extension before rapidly dropping to zero due to the mechanical constraint imposed by the model to prevent elbow hyperextension. In real lifts, however, a deceleration phase is observed during the final portion of the movement, reducing the mechanical loading acting on the joint structures [55].

### 4.3. Limitations

The primary limitation of this study concerns the values $\tau_{1}$ and $\tau_{2}$ used as inputs in direct dynamic numerical simulations. While we tested a broad range of input torque values to evaluate their effects on barbell kinematics, these values were kept constant throughout the entire range of motion. It is well established that the maximum muscle torque-generating capacity around a joint varies across the range of motion, mainly due to changes in muscle fiber length and shortening velocity, as well as muscle moment arm. A more accurate determination of $\tau_{1}$ and $\tau_{2}$ during maximal-effort multi-joint exercises would require an inverse dynamic approach combined with EMG measurements and musculoskeletal models incorporating key architectural parameters (e.g., force–length and force–velocity relationships, as well as moment arm variations as a function of joint angles). Nevertheless, due to muscle redundancy, such models rely on optimization methods [56,57] that are, to a large extent, arbitrary, potentially limiting the reliability of the results [22]. Moreover, this type of analysis is both conceptually and methodologically far beyond the intended scope of this present study. By contrast, assuming sets of constant values for $\tau_{1}$ and $\tau_{2}$ across the range of motion allowed us to isolate and analyze the differential contributions of the shoulder and elbow torques across distinct phases of the movement, which aligns precisely with the objective of this research.

## 5. Conclusions

In conclusion, this study provides a comprehensive biomechanical modeling of the trunk and upper limbs during the barbell bench press and overhead press, emphasizing the critical role of joint torques and the constraints imposed by the barbell in shaping movement dynamics. The analytical derivation of system equations and reaction forces enabled a detailed examination of the conditions leading to the sticking region, revealing how variations in shoulder and elbow torques influence barbell acceleration and deceleration phases. The direct dynamic simulations confirmed these findings, closely aligning with the experimental data from previous studies. While the assumption of constant joint torques represents a methodological limitation, it facilitated the isolation of key biomechanical factors governing lift performance. Future research incorporating musculoskeletal modeling and inverse dynamics could further refine our understanding of muscle activation strategies in upper-limb, multi-joint movements.

## Data Availability

The raw data supporting the conclusions of this article will be made available by the authors on request.

## Appendix A

Equation (A1) provides the following relationships expressing the trigonometric function of $\theta_{2}$ in terms of those of $\theta_{1}$:

(A1) $$\left\{\begin{array}{l} \cos\theta_{2}=\frac{d}{l_{2}}-\frac{l_{1}}{l_{2}}\cos\theta_{1}\\ \sin\theta_{2}=\sqrt{1-{\left(\frac{d}{l_{2}}-\frac{l_{1}}{l_{2}}\cos\theta_{1}\right)}^{2}}\\ \sin\left(\theta_{2}-\theta_{1}\right)=\cos\theta_{1}\sqrt{1-{\left(\frac{d}{l_{2}}-\frac{l_{1}}{l_{2}}\cos\theta_{1}\right)}^{2}}-\sin\theta_{1}\left(\frac{d}{l_{2}}-\frac{l_{1}}{l_{2}}\cos\theta_{1}\right)\\ \cos\left(\theta_{2}-\theta_{1}\right)=\cos\theta_{1}\left(\frac{d}{l_{2}}-\frac{l_{1}}{l_{2}}\cos\theta_{1}\right)+\sin\theta_{1}\sqrt{1-{\left(\frac{d}{l_{2}}-\frac{l_{1}}{l_{2}}\cos\theta_{1}\right)}^{2}} \end{array}\right.$$

The accelerations of J_1_, J_2_, C_1_, and C_2_ are determined by the basic equations of rigid body kinematics:

(A2) $$\left\{\begin{array}{l} {\vec{a}}_{J_{1}}={\ddot{\theta }}_{1}l_{1}{\hat{w}}_{1}-{\dot{\theta }}_{1}^{2}l_{1}{\hat{u}}_{1}\\ {\vec{a}}_{C_{1}}={\ddot{\theta }}_{1}l_{C_{1}}{\hat{w}}_{1}-{\dot{\theta }}_{1}^{2}l_{C_{1}}{\hat{u}}_{1}\\ {\vec{a}}_{J_{2}}={\vec{a}}_{J_{1}}+\left({\vec{a}}_{J_{2}}-{\vec{a}}_{J_{1}}\right)=\left({\ddot{\theta }}_{1}l_{1}{\hat{w}}_{1}-{\dot{\theta }}_{1}^{2}l_{1}{\hat{u}}_{1}\right)+\left({\ddot{\theta }}_{2}l_{2}{\hat{w}}_{2}-{\dot{\theta }}_{2}^{2}l_{2}{\hat{u}}_{2}\right)\\ {\vec{a}}_{C_{2}}={\vec{a}}_{J_{1}}+\left({\vec{a}}_{C_{2}}-{\vec{a}}_{J_{1}}\right)=\left({\ddot{\theta }}_{1}l_{1}{\hat{w}}_{1}-{\dot{\theta }}_{1}^{2}l_{1}{\hat{u}}_{1}\right)+\left({\ddot{\theta }}_{2}l_{C_{2}}{\hat{w}}_{2}-{\dot{\theta }}_{2}^{2}l_{C_{2}}{\hat{u}}_{2}\right) \end{array}\right.$$
